# Supplementary material for: Unravelling reference bias in ancient DNA datasets
Source: Bioinformatics. 2024 Jul 3;40(7):btae436. doi: 10.1093/bioinformatics/btae436 (PMC11254355; doi:10.1093/bioinformatics/btae436)
Supplement: btae436_Supplementary_Data [file btae436_supplementary_data.zip › S1-S4_data_plots.zip!/S1-S4_data/elephant_sim_plots.zip!/elephant_sim_plots/local_bowtie_elephant_div4_50end_by_MQ.pdf]

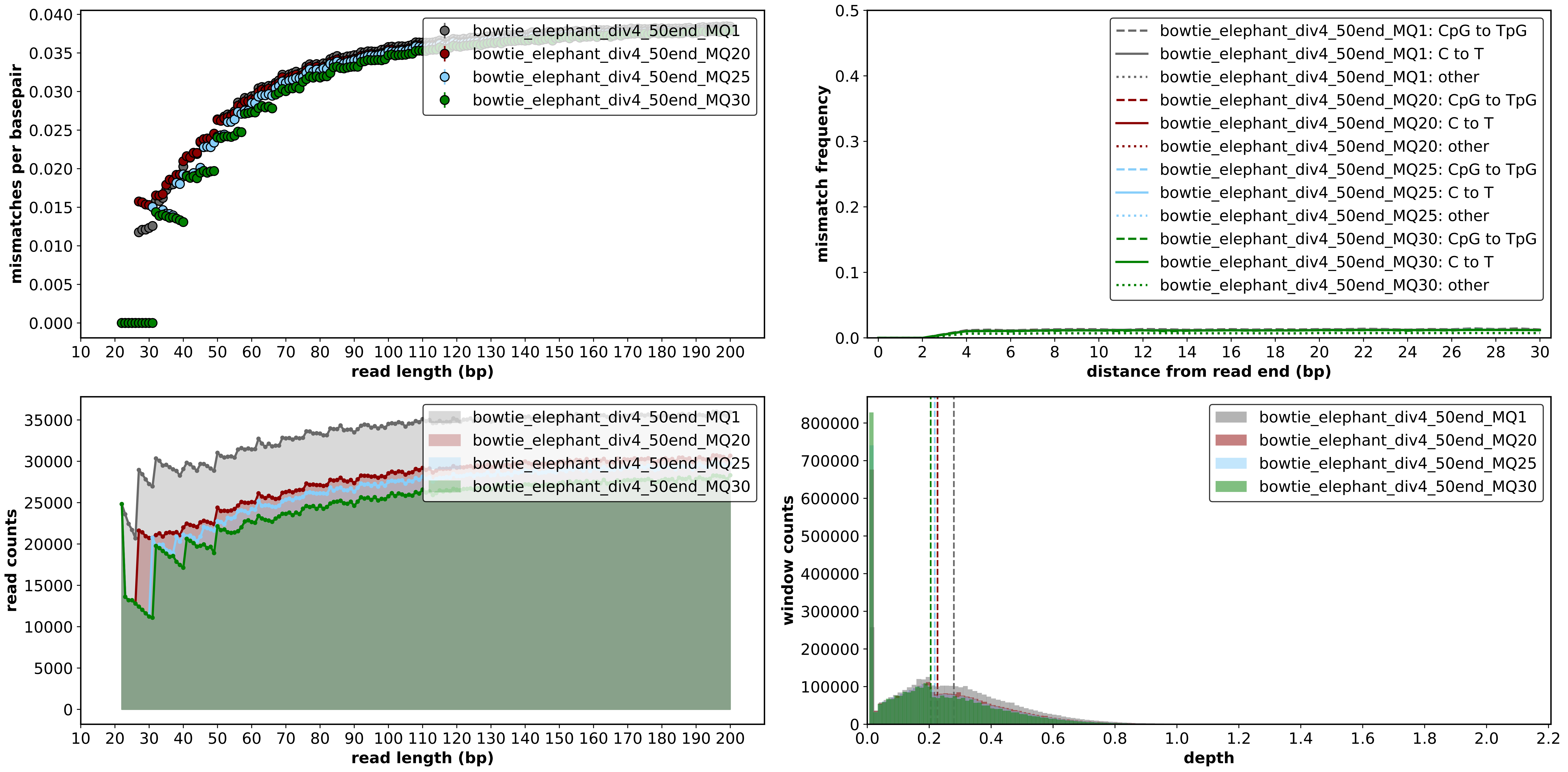

0.040
0.035
0.030
0.025
0.020
0.015
0.010
0.005
0.000
mismatches per basepair
10 20 30 40 50 60 70 80 90 100 110 120 130 140 150 160 170 180 190 200
read length (bp)
bowtie_elephant_div4_50end_MQ1
bowtie_elephant_div4_50end_MQ20
bowtie_elephant_div4_50end_MQ25
bowtie_elephant_div4_50end_MQ30
0.5
0.4
0.3
0.2
0.1
0.0
mismatch frequency
0 2 4 6 8 10 12 14 16 18 20 22 24 26 28 30
distance from read end (bp)
bowtie_elephant_div4_50end_MQ1: CpG to TpG
bowtie_elephant_div4_50end_MQ1: C to T
bowtie_elephant_div4_50end_MQ1: other
bowtie_elephant_div4_50end_MQ20: CpG to TpG
bowtie_elephant_div4_50end_MQ20: C to T
bowtie_elephant_div4_50end_MQ20: other
bowtie_elephant_div4_50end_MQ25: CpG to TpG
bowtie_elephant_div4_50end_MQ25: C to T
bowtie_elephant_div4_50end_MQ25: other
bowtie_elephant_div4_50end_MQ30: CpG to TpG
bowtie_elephant_div4_50end_MQ30: C to T
bowtie_elephant_div4_50end_MQ30: other
35000
30000
25000
20000
15000
10000
5000
0
read counts
10 20 30 40 50 60 70 80 90 100 110 120 130 140 150 160 170 180 190 200
read length (bp)
bowtie_elephant_div4_50end_MQ1
bowtie_elephant_div4_50end_MQ20
bowtie_elephant_div4_50end_MQ25
bowtie_elephant_div4_50end_MQ30
800000
700000
600000
500000
400000
300000
200000
100000
0
window counts
0.0 0.2 0.4 0.6 0.8 1.0 1.2 1.4 1.6 1.8 2.0 2.2
depth
bowtie_elephant_div4_50end_MQ1
bowtie_elephant_div4_50end_MQ20
bowtie_elephant_div4_50end_MQ25
bowtie_elephant_div4_50end_MQ30
